# A three-step approach for the derivation and validation of high-performing predictive models using an operational dataset: congestive heart failure readmission case study

**DOI:** 10.1186/1472-6947-14-41

**Published:** 2014-05-27

**Authors:** Samir E AbdelRahman, Mingyuan Zhang, Bruce E Bray, Kensaku Kawamoto

**Affiliations:** 1 Department of Biomedical Informatics, University of Utah, 615 Arapeen Way, Suite 208, Salt Lake City, UT 84092, USA; 2 Computer Science Department, Faculty of Computers and Information, Cairo University, Cairo, Egypt; 3 Departments of Biomedical Informatics and Internal Medicine, University of Utah, Salt Lake City, UT 84092, USA

**Keywords:** Predictive analytics, Congestive heart failure readmission, Voting classifiers, Feature selection, Discretization method, Feature ranking strategies

## Abstract

**Background:**

The aim of this study was to propose an analytical approach to develop high-performing predictive models for congestive heart failure (CHF) readmission using an operational dataset with incomplete records and changing data over time.

**Methods:**

Our analytical approach involves three steps: pre-processing, systematic model development, and risk factor analysis. For pre-processing, variables that were absent in >50% of records were removed. Moreover, the dataset was divided into a validation dataset and derivation datasets which were separated into three temporal subsets based on changes to the data over time. For systematic model development, using the different temporal datasets and the remaining explanatory variables, the models were developed by combining the use of various (i) statistical analyses to explore the relationships between the validation and the derivation datasets; (ii) adjustment methods for handling missing values; (iii) classifiers; (iv) feature selection methods; and (iv) discretization methods. We then selected the best derivation dataset and the models with the highest predictive performance. For risk factor analysis, factors in the highest-performing predictive models were analyzed and ranked using (i) statistical analyses of the best derivation dataset, (ii) feature rankers, and (iii) a newly developed algorithm to categorize risk factors as being strong, regular, or weak.

**Results:**

The analysis dataset consisted of 2,787 CHF hospitalizations at University of Utah Health Care from January 2003 to June 2013. In this study, we used the complete-case analysis and mean-based imputation adjustment methods; the wrapper subset feature selection method; and four ranking strategies based on information gain, gain ratio, symmetrical uncertainty, and wrapper subset feature evaluators. The best-performing models resulted from the use of a complete-case analysis derivation dataset combined with the Class-Attribute Contingency Coefficient discretization method and a voting classifier which averaged the results of multi-nominal logistic regression and voting feature intervals classifiers. Of 42 final model risk factors, discharge disposition, discretized age, and indicators of anemia were the most significant. This model achieved a c-statistic of 86.8%.

**Conclusion:**

The proposed three-step analytical approach enhanced predictive model performance for CHF readmissions. It could potentially be leveraged to improve predictive model performance in other areas of clinical medicine.

## Background

Hospital readmission is an admission to a hospital following an initial hospitalization. A common readmission timeframe measured by organizations such as the Centers for Medicare and Medicaid Services (CMS) is readmissions within 30 days of the index hospitalization [1]. Readmissions are considered undesirable clinical outcomes because they suggest that the patient was discharged prematurely from the initial hospitalization or that the post-hospitalization care was sub-optimal. In 2009, Jencks *et al.* reported that of 11.9 million Medicare beneficiaries discharged from a U.S. hospital within a 15-month period from 2003 to 2004, 19.6% of the patients were readmitted within 30 days, with unplanned hospitalizations leading to $17.4 billion in excess costs to Medicare in 2004 [2]. Moreover, an index visit for congestive heart failure (CHF) was followed by a readmission in 26.9% of cases in this study, with CHF representing the most common reason for an index visit leading to a readmission. The CMS Readmissions Reduction Program provides a financial incentive for hospitals to reduce readmissions, as high rates of readmissions for CHF and several other conditions can lead to an assessment of financial penalties to hospitals [1].

Given the importance of readmission both clinically and financially, there have been significant efforts to identify individuals at elevated risk of readmission, so that they can be targeted for interventions aimed at reducing readmissions. Recently, predictive analytics has emerged as an effective method for identifying patients at elevated risk of readmissions [3-17]. To improve their performance, some readmission predictive models have used statistical and/or classification techniques to analyze the candidate explanatory variables and to select those variables that should be included as risk factors in the final predictive model. To our knowledge, however, the wrapper subset feature selection method, which has been suggested to be a superior approach to feature selection [18-20], has not been applied in the area of readmission predictive analytics.

As with any predictive model, the main indicator of model performance is the c-statistic, which is equivalent to the area under the curve (AUC) of the receiver operating characteristic (ROC) plot. To our knowledge, the highest performing predictive models for all-cause readmissions have associated c-statistic values of 77.1% [16] and 83.3% [17], and for CHF readmission, < 80% [21-35]. Because a predictive model should have a c-statistic ≥ 80% to be considered good, the lack of such a predictive model for CHF readmissions presents a challenge for predicting and addressing CHF readmissions.

Predictive models for readmissions are typically developed using clinical and administrative data collected as a part of the care process. Unfortunately, such operational clinical data frequently contain incomplete patient records [36]. This issue may be adjusted using several methods [37]. Two of the most common approaches to adjusting for missing data are complete-case analysis and mean-based imputation. In complete-case analysis, incomplete data records are simply removed from the dataset. In mean-based imputation [38], missing values are filled using the mean/mode of the completed values or values determined using K-means algorithms. There is no universally optimal method for handling missing data, since each dataset has unique characteristics [37]. Therefore, each study should evaluate alternate approaches to adjusting for missing data and select the approach with the best performance for that dataset.

In addition to incomplete records, an additional challenge of operational datasets is that such data may change over time, e.g., due to changes in clinical practice or the introduction of a new health information system. Therefore, it is unclear whether selecting a single timeframe for model development is adequate [10].

In recent years, biomedical researchers have investigated the use of voting classifiers [39-41] and discretization algorithms [42,43] to enhance the performance of classification methods. Voting classifiers enhance performance by combining (weighting) the outputs of two or more classifiers, while discretization enhances classification performance by converting numeric variables (e.g., age) into categorical variables (e.g., age 0–20 versus age 21–25, etc.) based on the data distribution.

In making use of a predictive model, it is often important to know which risk factors are most significant. For example, knowing which clinical factors are most significant for predicting CHF readmissions is important, because it allows for more targeted intervention on those factors. Such risk factor identification is often accomplished using statistical measures (e.g., p-values and odds ratios). However, it has been suggested that other feature selection and ranking strategies are superior [18,20]. These potentially superior methods include the wrapper subset feature selection method, as well as ranking strategies based on information gain, gain ratio, symmetrical uncertainty, and wrapper subset feature evaluators.

In this manuscript, we propose a systematic, three-step approach to healthcare predictive analytics that (i) accounts for the changing and frequently incomplete nature of operational clinical data, (ii) empirically develops optimal predictive models using a combination of various statistical analyses, adjustment methods for handling missing values, feature selection methods, classifiers, and discretization methods, and (iii) empirically categorizes each risk factor as being strong, regular, or weak using a combination of statistical analyses, feature rankers, and a newly developed ranking algorithm.

This systematic, three-step approach to predictive analytics was evaluated in the context of predicting CHF readmission within a tertiary academic medical center. To our knowledge, our proposed three-step methodology for optimizing predictive analytics has not been applied to any healthcare domain to date. Here, we summarize the methods and results from this research and discuss the implications, limitations, and future direction of our work.

## Methods

### Subjects and settings

This study was conducted at University of Utah Health Care (UUHC), which is a tertiary academic health system centered in Salt Lake City, Utah. This study was approved by the University of Utah Institutional Review Board (Protocol # 00060215).

The subjects evaluated were individuals hospitalized for CHF at UUHC and admitted between January 1st 2003 and June 30th 2013. CHF hospitalizations were defined as those hospitalizations with a primary discharge diagnosis of one of the following ICD9 codes: 402.01, 402.11, 402.91, 404.01, 404.03, 404.11, 404.13, 404.91, 404.93, or 428.XX. This research analyzed 2,787 hospitalizations matching these criteria unless the hospitalization was missing a required explanatory variable as described below. The most frequent primary discharge diagnoses were unspecified CHF (ICD9 428.0, 41.98%), systolic CHF (ICD9 428.2X, 33.62%), and diastolic CHF (ICD9 428.3X, 14.03%).

### Dataset

The data utilized for this analysis was obtained from the UUHC Enterprise Data Warehouse. For each of the 2,787 CHF hospitalizations from January 1st 2003 to June 30th 2013, a number of potential explanatory variables were obtained based on their use in the prior literature in the field [24,33] and their availability in the data warehouse. The data analyzed included demographic information; data on the index hospitalization, such as discharge disposition, comorbidities, laboratory tests, and vital signs; and healthcare utilization during the 6 months prior to the hospitalization. Whether an index hospitalization was followed by a readmission within 30 days was captured as an attribute of the index hospitalization.

#### Missing data

Vital sign data began to be available regularly from 2008, when a new electronic health record (EHR) system was implemented at UUHC. Given the importance of vital sign data in prior literature in this field, the availability of vital sign data was an important consideration for the definition of the model derivation datasets, as described below. In addition to vital signs, certain laboratory tests of interest were present in only a minority of encounters.

#### Model derivation and validation datasets

In order to account for the significant difference in vital sign availability before and after 2008, three candidate datasets for model derivation were generated: a dataset containing all years except the validation year (2003–2012); a dataset containing the transition year for vital sign data and subsequent years (2008–2012); and a dataset containing years following the transition year (2009–2012). In addition, a validation dataset was generated for hospitalizations from the first six months of 2013. The numbers of records are as follows: 1122 (5.34% complete) for 2003–2007, 227 (42.73% complete) for 2008, 1250 (47.36% complete) for 2009–2012, and 188 (47.34% complete) for the first six months of 2013. The annual readmission rates for 2003 through the first six months of 2013 were 17.06%, 14.50%, 15.38%, 24.11%, 11.81%, 10.57%, 16.78%, 23.20%, 19.94%, 15.50%, and 8.50% respectively. Because there was no clear trend in readmission rates over time, readmission rates were not used to define the temporal derivation datasets.

To handle the missing values, we evaluated the complete-analysis adjustment method and all mean-based imputation algorithms described by Luengo *et al.*[38]. However, as these imputation methods resulted in the development of models with inferior predictive ability with regard to AUC, positive predictive value (PPV), and negative predictive value (NPV), our decision was to use only complete records for our derivation datasets. We defined a complete record to be one which contained all the features considered for the model. The final candidate derivation datasets therefore consisted of hospitalizations with complete data from 2003–2012, 2008–2012 and 2009–2012, with total record counts of 749, 689, and 592 respectively. The validation dataset encompassed all 188 available hospitalizations, including hospitalizations with incomplete data. All model validations were conducted using the full validation dataset, including the records with missing data.

### Dependent and independent variables

The *dependent variable* for the study was readmission (repeat inpatient hospitalization) for any cause within 30 days of the index CHF hospitalization. A given hospitalization could serve both as a readmission to an earlier index CHF hospitalization, as well as the index CHF hospitalization for a later readmission. We considered but did not use the CMS definition for CHF readmission [31], because this more restrictive definition would have reduced the available sample size for the analysis. The *independent explanatory variables* and their acronyms are summarized in Table 1 and are described below.

**Table 1:** Independent explanatory variables

**Variable**	**Values**
**Gender**	Female, Male
**Religion**	Assembly Of God, Atheist, Baptist, Buddhist, Catholic, Christian, Church Of Christ, Episcopalian, Greek Orthodox, Islamic, Jehovah's Witness, Jewish, Latter Day Saints, Lutheran, Methodist, Missing, Muslim, Native, No spiritual preference/needs, Non-Denominational, Not Verified, Pentecostal, Presbyterian, Protestant, Seventh Day Adventist, Spiritual, Not Religious, Unable To Answer, Unitarian, Other
**Marital status**	Divorced, Legally Separated, Life/Domestic Partner, Married, Single, Unknown, Widowed
**Race**	African American, American Indian-Alaska, Asian, Hawaiian-Pacific Islander, Missing, Others, Patient Refused, Unknown, White Or Caucasian
**Hospital service**	Bone Marrow Transplant, Cardiology, Cardiothoracic Surgery, Emergency, General Surgery, Hematology/Oncology, Internal Medicine, Nephrology, Observation, Pulmonology, Rheumatology, Transplant Study, Vascular Surgery
**Discharge disposition**	Dsch/Xfer Court/Law Enforcement, Expired, Federal Hospital, Home Health Care Svc, Home Or Self Care, Hospice/Homem, Hospice/Medical Facility, Intermediate Care Facility, Left Against Medical Advice, Long Term Care, Psychiatric Hospital, Rehab Facility, Skilled Nursing Facility
**Insurance/Finance Class**	Agencies, Champus, Commercial, Facility, Grants&Studies, Medicaid, Medicare, Pehp, Self Pay, UT Misc Government, UT Workers Comp, Healthcare Network
**Charlson Index Frequency (CharlsonIndexF)**	Numeric values
**Vital signs:**	Numeric values
**First Reading Weight KG (FW)**	
**Last Reading Heart Rate (LHR)**	
**Last Reading Systolic Blood Pressure (LSBP)**	
**Last Reading Weight KG (LW)**	
**Age at admission (AGE)**	Numeric values
**Zip code**	Numeric values
**Length of Stay (LOS)**	Numeric values
**Prior 6-month: Emergency Department Frequency (PriorEDF)**	Numeric values
**Prior 6-month: Mean CharlsonIndex Frequency (PriorCharlsonIndexF)**	Numeric values
**Prior 6-month: Mean Emergency Department Length of Stay (PriorEDLOS)**	Numeric values
**Clinical Classifications Software (CCS) Category for ICD-9-CM**	Numeric values
**1 Infectious and parasitic diseases**	
**2 Neoplasms**	
**3 Endocrine, nutritional and metabolic diseases, and immunity disorders**	
**4 Diseases of the blood and blood-forming organs**	
**5 Mental disorders**	
**6 Diseases of the nervous system and sense organs**	
**7 Diseases of the circulatory system**	
**8 Diseases of the respiratory system**	
**9 Diseases of the digestive system**	
**10 Diseases of the genitourinary system**	
**11 Complications of pregnancy, childbirth, and the puerperium**	
**12 Diseases of the skin and subcutaneous tissue**	
**13 Diseases of the musculoskeletal system and connective tissue**	
**14 Congenital anomalies**	
**15 Certain conditions originating in the perinatal Period**	
**16 Injury and poisoning**	
**17 Symptoms; signs; and Ill-defined conditions and factors influencing health status**	
**18 Residual codes; unclassified; all E codes**	
**32 Variables of laboratory tests:**	Numeric values
**Alanine Aminotransferase(ALT)-U/L**	
**Albumin, Serum or Plasma(ALBUMIN)-g/dL**	
**Alkaline Phosphatase (ALKPHOS)-U/L**	
**Aspartate Aminotransferase(AST)-U/L**	
**Basophil #(BASO)-k/uL**	
**Basophil %(BASO%)-%**	
**Bilirubin, total(BILIRUBIN)-mg/dL**	
**B-Type Natriuretic Peptide (BNP)-pg/mL**	
**Creatinine, Serum or Plasma(CREAT)-mg/dL**	
**Eosinophil #(EOSIN)-k/uL**	
**Eosinophil %(EOSIN%)-%**	
**Glucose, serum or plasma(GLUC)-mg/dL**	
**Granulocyte #(GRANULO)-k/uL**	
**Granulocyte %(GRANULO%)-%**	
**Hematocrit(HCT)-%**	
**Hemoglobin(HGB)-g/dL**	
**Lymph #(LYMPHO)-k/uL**	
**Lymphocyte %(LYMPHO%)-%**	
**Mean Corpuscular Hemoglobin(MCHGB)-pg**	
**Mean Corpuscular Hgb Concentration(MCHGBCON)-g/dL**	
**Mean Corpuscular Volume (MCVOL)-fL**	
**Mean Platelet Volume(PLATVOL)-fL**	
**Monocyte #(MONO)-k/uL**	
**Monocyte %(MONO%)-%**	
**Platelet(Platelet)-k/uL**	
**Potassium, Serum or Plasma(POTASS)-mmol/L**	
**Protein, total, serum or plasma(PROTEIN)-g/dL**	
**Red Blood Cell(RBC)- M/uL**	
**Red Cell Distribution Width(RCDW)-%**	
**Sodium, Serum or Plasma(SODIUM)-mmol/L**	
**Urea Nitrogen, Serum or Plasma(UREANIT)-mg/dL**	
**White Blood Cell Count(WBC)-k/uL**	

Explanatory variables are noted in bold and their values are noted using a normal font. The acronyms of the variables are listed in bold parentheses. The lab variable is presented in the format of name (acronym)-measure unit. The table includes the gender, religion, discharge disposition, marital status, insurance/finance class, and hospital service. It also includes the laboratory tests, Clinical Classification Software (CCS) category for ICD-9-CM diagnoses, Charlson Index frequency (CharlsonIndexF), vital sign variables, age at admission, zip code, length of stay (LOS), and the prior 6-month variables of emergency department frequency, mean Charlson Index frequency, and mean length of stay in the emergency department.

*Demographic information* included gender, race, religion, marital status, insurance/finance class, age, and the zip code of the home address. From the zip code, home proximity and mean household income were estimated as follows: (i) obtain longitude and latitude from the U.S. Census Bureau [44]; (ii) calculate home proximity from UUHC using the Haversine formula [45]; and (iii) obtain mean household income based on the zip code [46].

*Hospitalization data* included discharge disposition, the responsible hospital service, and the length of stay (LOS). Also, each index hospitalization had from 3 to 62 ICD9 billing diagnoses. These *comorbidities* were abstracted as follows. First, the number of comorbid conditions included in the Charlson index for comorbidities [47] was identified and abstracted as the CharlsonIndexF variable. Then, the codes not included in this variable were classified into the 18 top-level diagnosis categories in the Agency for Healthcare Research and Quality’s Clinical Classifications Software (CCS) system (Table 1), and the count of codes in each category was calculated [48].

We were originally interested in 48 *laboratory tests* based on their suggestion in the literature as well as the clinical judgment of a practicing cardiologist (BEB). Sixteen tests were excluded because of missing values in > 50% of cases. Only those tests listed in Table 1 were included. *Vital signs* included 4 variables: last reading systolic blood pressure (LSBP), last heart rate (LHR), first reading weight (FW), and last reading weight (LW). In all vital signs, the first reading refers to the first reading upon hospital admission and the last reading refers to the last reading prior to discharge.

*Pre-hospitalization information* from the 6 months prior to the index hospitalization included the frequencies of Charlson index comorbidities from any type of encounter (i.e., outpatient, emergency department [ED], or inpatient), captured as the PriorCharlsonIndexF variable. Also, we included the number of ED encounters (PriorEDF) and the mean of corresponding lengths of stay (PriorEDLOS).

Age, LOS, CharlsonIndexF, comorbidities, vital signs, laboratory tests, proximity and income are numeric variables, and the others are categorical.

### Proposed approach

#### Thresholds for statistical significance of variables

As noted in the introduction, feature selection based on the p-values of statistical tests is common in predictive analytics. Typically, variables with p-values ≤ 0.001 are considered significant risk factors, variables with p-values ≥ 0.1 are considered irrelevant, and variables with intermediate p-values are considered moderately significant. Throughout this research, we used these typical thresholds.

#### Overview of approach

**Figure 1:**
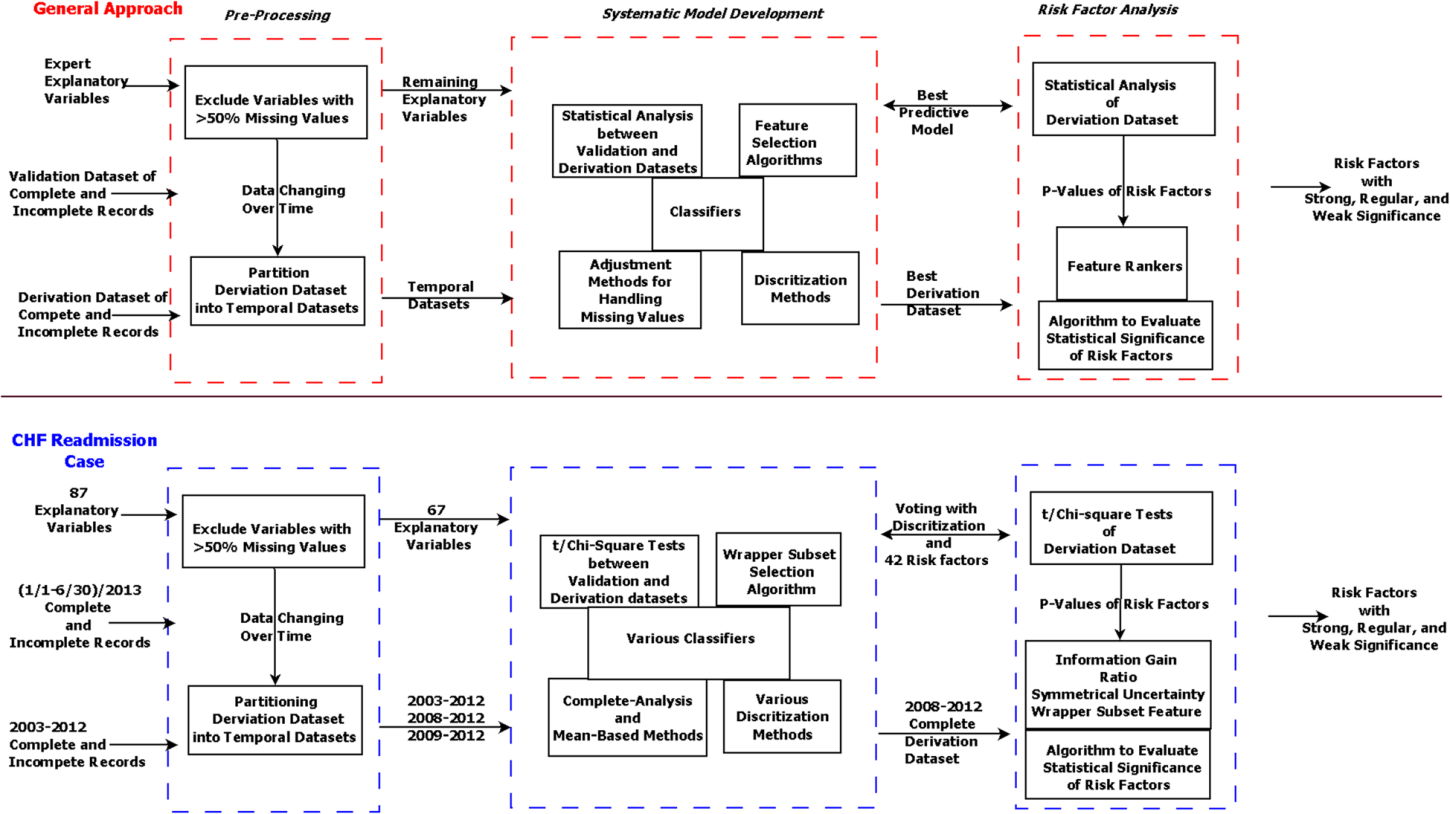
**Diagram of the proposed general approach and its implementation in the congestive heart failure (CHF) readmission case.** The upper part of the figure is the proposed general approach steps and the lower part is for their CHF readmission counterparts. The three steps of the approach, namely pre-processing, systematic model development, and risk factor analysis, are shown in italic-bold cases and are bounded by red and blue lines with shaded backgrounds for the proposed general approach and CHF readmission case study respectively.

Figure 1 provides a graphical overview of the proposed three-step approach and its application to the CHF readmission case study. As the first step, the data is *pre-processed*, with explanatory variables missing in 50% or more of cases being excluded. Also, the changing nature of operational data over time is addressed by generating different derivation datasets based on key time-points during which changes occurred to the underlying data. Then, in the second step, *systematic model development* is undertaken. Here, the most appropriate classifier, features, discretization algorithm, adjustment method for missing data, and derivation dataset are selected in order to develop the highest performing predictive models. Finally, as the third step, *risk factor analysis* is conducted on the final predictive model in order to rank the risk factors in terms of their relative significance. Provided below are details of these steps in the proposed approach.

#### First step: pre-processing

The input of this step is the explanatory variables identified by experts and from the literature, as well as the validation and derivation datasets containing both complete and incomplete records. This step analyzes the full dataset (2003 to 2013 in our case study), and variables with ≥ 50% missing information are removed. Also, the distributions of categorical variable values over time are examined, so as to identify changes in data collection practices and to account for such changes. In particular, if there are key points in time when data collection practices or underlying data changes occurred, temporal datasets are generated for model derivation purposes based on these key time points. Furthermore, categorical values are merged if they changed over time to represent the same concept. For example, in this case study, the introduction of a new scheduling and billing system changed the designation of the cardiothoracic surgery service from the “CTI” service to the “CTS” service. Such equivalent categorical values are merged into a single variable (e.g., “Cardiothoracic Surgery” in this case). All categorical values are otherwise used without modification. The outputs of this step are the remaining explanatory variables and the candidate temporal datasets.

#### Second step: systematic model development

The inputs of this step are the outputs from the first step. This step aims to develop the highest performing models through the selection of the most appropriate classifier, features, discretization algorithm, adjustment method for missing data, and derivation dataset. The outputs from this step are the highest performing predictive model and the associated derivation dataset and methods.

This step begins with preliminary statistical analyses to characterize the data. To study the variable distributions in the derivation datasets compared to the validation dataset, statistical tests are used to identify the closeness of the variable distribution in each derivation dataset compared to the validation dataset. For this analysis, the dependent variable is whether the data is from a derivation dataset (value = yes) or is from the validation dataset (value = no). Then, each candidate derivation dataset is combined with the validation dataset, wherein the independent variables are the independent variables following pre-processing, as well as the outcome of interest (for the case study, whether the CHF hospitalization was followed by a readmission). Then, χ^2^ and t-tests are used for categorical and numeric variables respectively to calculate the related p-values. We use these tests to identify which derivation dataset(s) are strongly correlated (p-value ≤ 0.001) with the validation dataset with regard to readmissions, as such correlation provides an initial indication of which derivation dataset(s) may serve as good candidate(s) for developing the final models. These tests were also used to identify whether any explanatory variable has highly significant p-values across the derivation datasets (defined as p-value > 0.1 vs. p-value ≤ 0.001), which would indicate a potential problem with the explanatory variable in the datasets that would require investigation or exclusion from further analysis.

Following the above analysis, the next step is to empirically identify the combination of classifier, features, discretization algorithm, and derivation dataset with the highest predictive ability, as measured by the c-statistic (AUC), accuracy (i.e., the percentage of true classifications with respect to all classification results), sensitivity, specificity, PPV, and NPV of the predictive model when tested against the validation dataset. For evaluating these metrics, a cutoff probability of 0.5 is used for predicting the outcome. In other words, if the probability of the outcome (in this case, readmission) is greater than 0.5, the prediction is for the outcome to occur; if it is less than or equal to 0.5, the prediction is for the outcome to not occur. The dependent variable in this analysis is the outcome of interest (in this case, readmission for any cause within 30 days of the index CHF hospitalization). For this analysis, all classifiers reviewed by [6,10,13,15,33,49] were assessed, as well as all pair-wise combinations of these classifiers (via voting). These classifiers were selected because they are typically used in the literature. These standard algorithms are supported in Weka 3.6 [50], and this open-source resource was utilized for this analysis. Each classifier was evaluated with the wrapper subset feature selection method and best fit strategy [20]. Furthermore, each of the discretization algorithms described by [42,43,51-53] was tested in combination with each classifier described above. For this purpose, the KEEL software tool [54] was utilized. Discretization is performed on numeric variables that are typically represented in terms of pre-defined intervals. In this case, age, LOS, home proximity, and mean household income were discretized in this manner. Accordingly, for each candidate derivation dataset, and for each classifier, there are predictive models with and without discretization. The highest-performing combination of factors is then empirically selected for the next step.

#### Third step: risk factor analysis

This step begins by restricting the analysis to the classifier, discretization algorithm, and derivation dataset identified as leading to the highest performance in the previous step. The objective of this step is to develop the final predictive model and to rank the most significant risk factors. The output of this step is the identification of risk factors in the final predictive model, with each risk factor ranked as strong, regular, or weak.

In terms of explanatory variables, in order to avoid omitting any significant variables, all variables resulting from the step one analysis are re-introduced. Then, the final explanatory variables are selected and ranked in the following manner. In this analysis, the dependent variable is whether the outcome of interest (in our case study, CHF readmission) exists (value = yes) or not (value = no). Then, using just the highest-performing derivation dataset, χ^2^ and t-tests are used for categorical and numeric variables respectively to identify the degree to which the variable is capable of discriminating between the outcomes of interest.

In this study, we used four ranking strategies based on information gain, gain ratio, symmetrical uncertainty, and wrapper subset feature evaluators [20]. Weka was leveraged to implement these strategies, with variables receiving relative weights totaling to 1. The variables are then ordered based on their relative weights using only those variables with a relative weight ≥ 0.001. Furthermore, using our own algorithm shown in Algorithm 1 risk factors are identified from the explanatory variables and categorized as strong, regular, or weak risk factors. While interactions among explanatory variables are considered for feature selection, in accordance with typical methodologies in this area [10,33], such interactions are not currently evaluated for the purposes of identifying the significance of risk factors. In the future, the approach proposed could be adapted to consider such interactions.

## Results

The proposed three-step approach to predictive analytics was applied to the prediction of CHF readmission using an operational dataset. Provided below are the results from this sample application of the proposed approach.

### Pre-processing step

**Figure 2:**
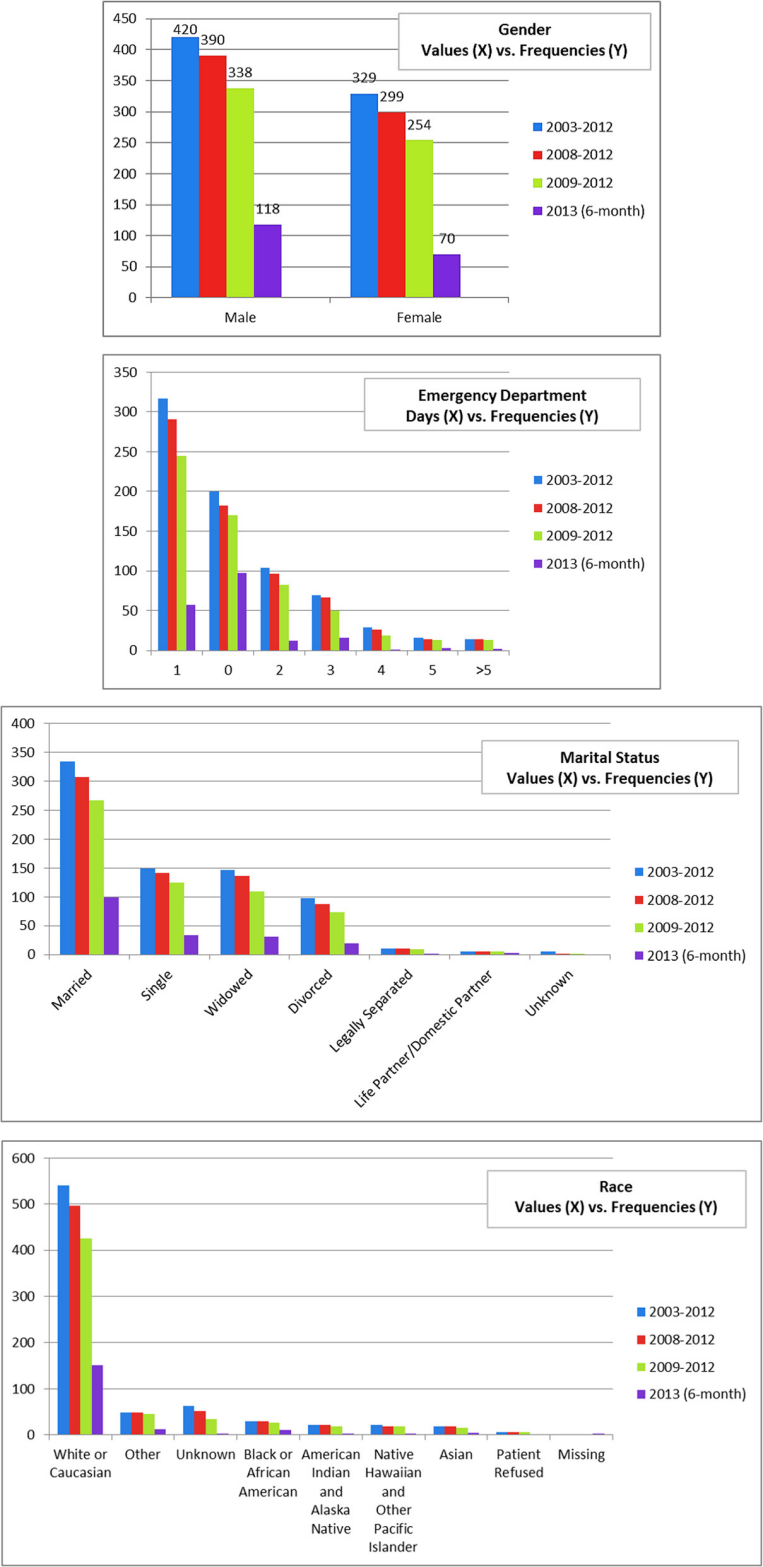
**Frequencies of the values of gender, emergency department, marital status, and race.** The datasets are from 2003–2012, 2008–2012, 2009–2012, and 2013 (6-month). The X axis stands for the values/days of the variable and the Y axis stands for the related frequencies.

**Figure 3:**
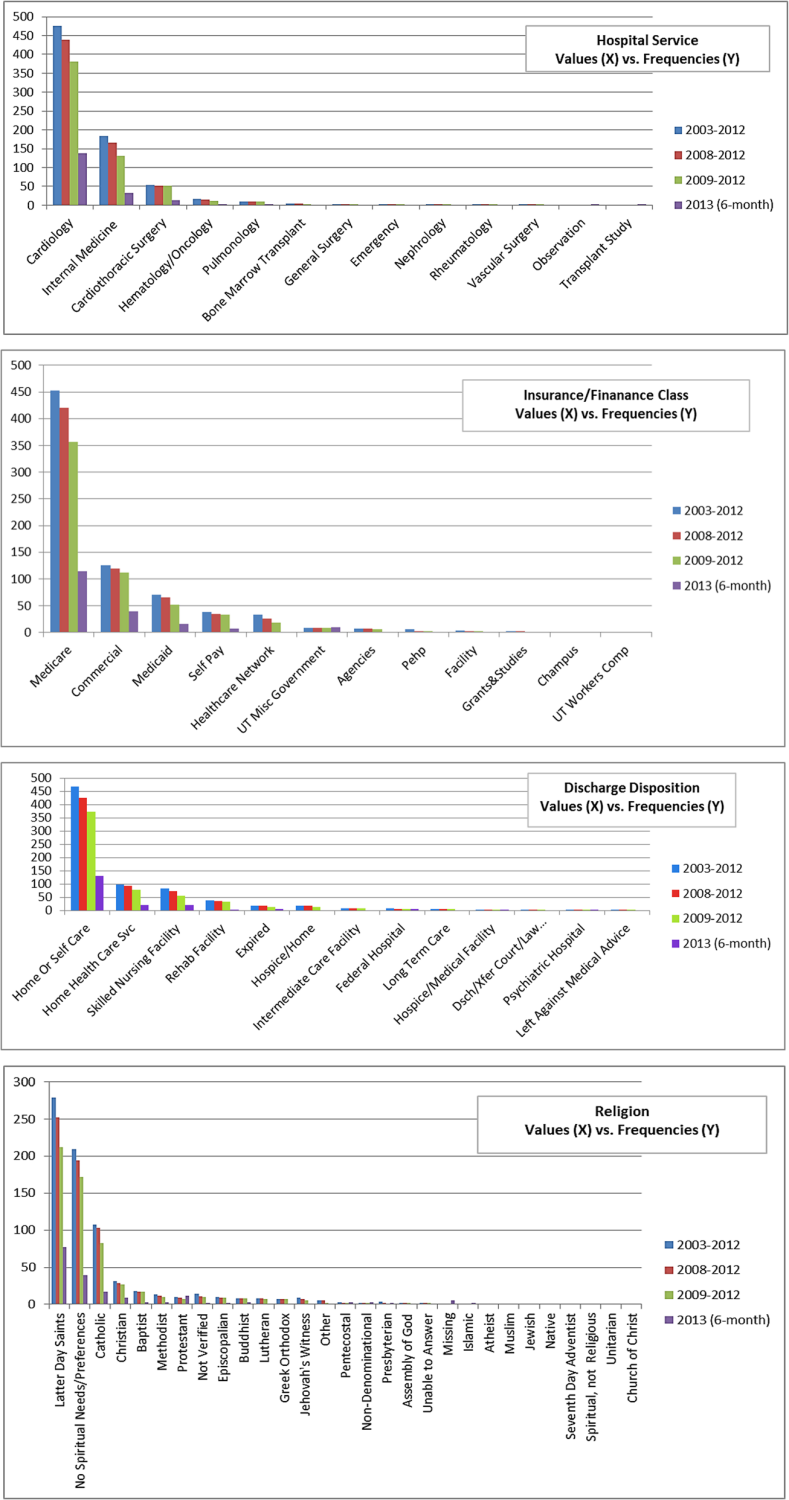
**Frequencies of the values of hospital service, insurance/finance class, discharge disposition, and religion.** The datasets are from 2003–2012, 2008–2012, 2009–2012, and 2013 (6-month). The X axis stands for the values of the variable and the Y axis stands for the related frequencies.

Of 83 original explanatory variables, 16 laboratory tests were removed due to > 50% missing information, resulting in 67 independent variables for analysis. Figures 2 and 3 describe the value distributions of the categorical variables. As noted, the gender variable was relatively equally distributed. However, the variables of marital status, race, religion, discharge disposition, hospital service, insurance/finance class, and PriorEDF were biased towards some specific values. Table 2 provides the mean and standard deviation (SD) of the numeric variables. Most numeric variables were clustered around their means except for LOS and home proximity, which had relatively high SDs due to many patients having high values.

**Table 2:** Correlation of derivation and validation datasets

	**2003-2012 vs. 2013 (6-month)**	**2008-2012 vs. 2013 (6-month)**	**2009-2012 vs. 2013 (6-month)**
**Gender**	0.099	0.135	0.175
**Race**	≤0.001	≤0.001	0.002
**Marital status**	0.406	0.499	0.632
**Religion**	≤0.001	≤0.001	≤0.001
**Insurance/Finance class**	≤0.001	≤0.001	0.009
**Age_At_Admission (Age)**	0.023	≤0.001	≤0.001
	(64.39/15.8)	(64.38/15.89)	(63.79/15.82)
**Home proximity**	0.011	≤0.001	≤0.001
	(93.2/205.64)	(93.65/206.78)	(95.73/196.36)
**Mean household income**	0.011	≤0.001	≤0.001
	(65650/19673.84)	(65630/19599.2)	(65580/19612)
**Discharge disposition**	0.02	0.01	0.01
**Hospital services**	0.19	0.236	0.43
**Length of Stay(LOS)**	≤0.001	≤0.001	≤0.001
	(7.46/9.16)	(7.56/9.33)	(7.73/196.36)
**CharlsonIndexF frequency**	≤0.001	≤0.001	≤0.001
	(4.87/2.84)	(4.87/2.83)	(4.92/2.89)
**18 CCS Categories**	≤0.001	≤0.001	≤0.001
	(0.062-4.94)/	(0.065-5.01)/	(0.069-5.03)/
	(0.270-2.55)	(0.277-2.55)	(0.287-2.6)
**Lab tests**	≤0.001	≤0.001	≤0.001
	(0.025-1430)/	(0.026-1456)/	(0.026-1446)/
	(0.047-130.57)	(0.047-130.41)	(0.049-132.82)
**4 Vital sign variables**	≤0.001	≤0.001	≤0.001
**LAST SBP (LSBP)**	(110.5/21.65)	(110.7/21.4)	(110.7/21.4)
**LAST HR (LHR)**	(79.71/15.92)	(79.88/15.98)	(79.77/15.91)
**FIRST WT KG(FW)**	(92.57/31.34)	(92.57/31.57)	(93.18/31.35)
**LAST WT KG(LW)**	(89.93/42.47)	(90.1/43.23)	(90.88/44.57)
**6-month Prior:**			
**Mean CharlsonIndexF**	≤0.001	≤0.001	≤0.001
**Frequency**	(2.99/7.5)	(3.04/7.615)	(3.08/7.7)
**ED Frequency (EDF)**	≤0.001	≤0.001	≤0.001
**Mean EDLOS**	≤0.001	≤0.001	≤0.001
	(0.16/0.17)	(0.15/0.13)	(0.15/0.13)
**Number of Yes/No records**	608/141	597/132	471/121
**Readmission**	0.0004	0.0002	0.0001

For each numeric variable, both the p-value and the related mean and standard deviation are provided. For laboratory tests and 18 CCS categories, the ranges of means with their associated standard deviations are listed. All variable acronyms are from Table 1.

### Systematic model development step

In this step, we used two adjustment methods, namely complete-case analysis and mean-based imputation based on algorithms described by Luengo *et al.*[38]. In complete-case analysis, incomplete data records are simply removed from the dataset. In mean-based imputation [38], missing values are filled using the mean/mode of the completed values or values determined using K-means algorithms. Of note, the tables and figures below refer to results utilizing the complete derivation datasets, since all models generated by complete-case analysis outperformed those of the mentioned imputation methods.

During the step one analysis (Table 2), all derivation datasets were strongly correlated (p-value ≤ 0.001) with the validation dataset with regard to readmissions, indicating that all the derivation datasets could potentially serve as good candidates for developing the final models. Moreover, the 2009–2012 and 2008–2012 datasets were most closely correlated with the validation dataset with regard to readmissions (p-value 0.0002 and 0.0001, respectively), suggesting that the highest model performance would likely be achieved using these datasets. Also, all p-values for the explanatory variables exhibited homogeneous variable distribution across the three derivation datasets, indicating that there was no need to investigate potential problems in the use of these explanatory variables at this stage.

**Figure 4:**
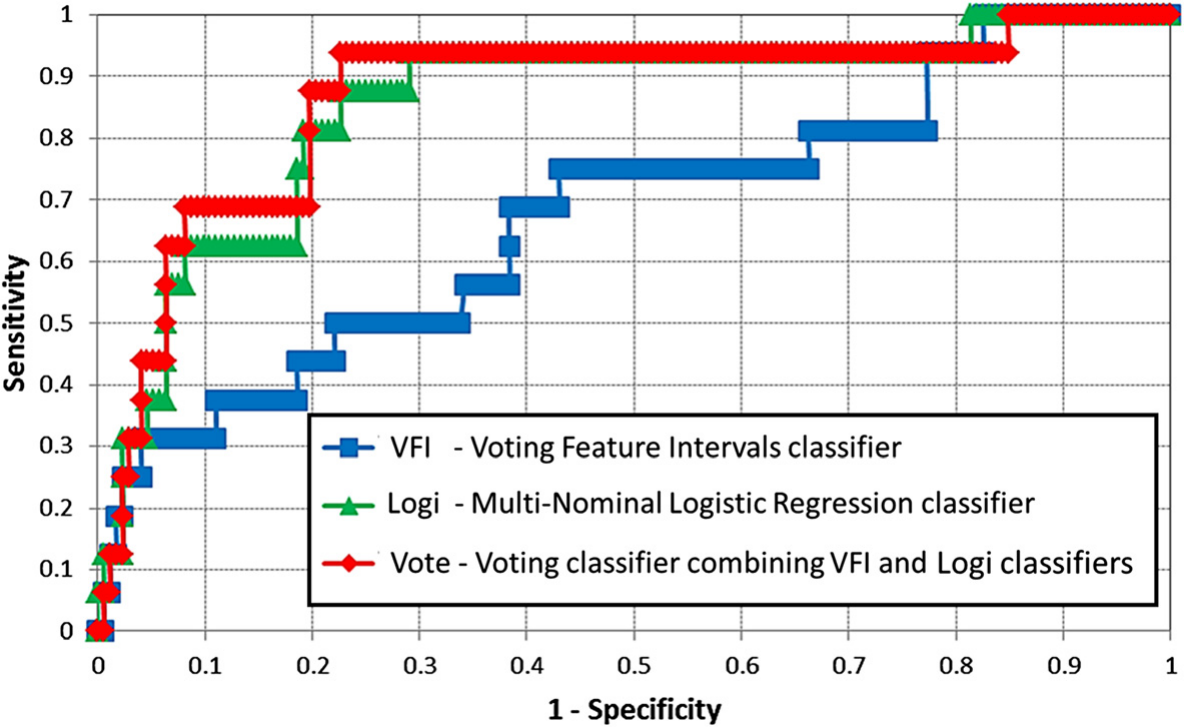
**AUC of the highest performing model and its component classifiers.** The derivation dataset was from 2008–2012 and 47 independent variables were used along with discretization.

Based on empirical analysis, the highest performing derivation dataset was from the complete 2008–2012 dataset. The performance characteristics of the best models from each of the candidate derivation datasets with and without discretization are shown in Table 3. The highest performing model resulted from utilizing a voting classifier that averaged the results of Multi-Nominal Logistic Regression [55] and Voting Feature Intervals (VFI) [56] classifiers along with the wrapper subset feature selection method and the Class-Attribute Contingency Coefficient Discretization (CACC-D) [53] algorithm. The model’s performance characteristics were 86.8%, 91.5%, 62.5%, 94.2%, 50%, and 96.4% for AUC, accuracy, sensitivity, specificity, PPV, and NPV respectively. Figure 4 shows the AUCs of the voting classifier and its counterparts. Of the 67 independent variables considered in this step, 42 were selected as candidate risk factors (variables in non-italic font in Tables 4 and 5).

**Table 3:** Performance characteristics of final models

	**AUC**	**Accuracy**	**Sens.**	**Spec.**	**PPV**	**NPV**
**2003-2012**						
**Without discretization**	80.3	87.23	43.8	91.3	31.82	94.58
**With discretization**	83	85.64	43.8	89.5	28	94.48
**2008-2012**						
**Without discretization**	**83.8**	**90.4**	**56.3**	**93.6**	**45**	**95.8**
**With discretization**	**86.8**	**91.5**	**62.5**	**94.2**	**50**	**96.4**
**2009-2012**						
**Without discretization**	83.5	86.7	62.5	89	38.46	96.23
**With discretization**	85	86.17	56.3	89	32.14	95.63

The models were developed using the strategy outlined in Figure 1. The results are shown for the three derivation datasets: 2003-2012, 2008-2012, and 2009-2012 without/with discretization; Sens. = sensitivity, spec. = specificity, PPV = positive predictive value, NPV = negative predictive value. The best results are indicated in bold rows.

**Table 4:** Final status of categorical and accumulated discretized explanatory variables

	**P-values**	**Main values**	**No (557)**	**Yes (132)**	**G/W**	**Inf**	**Sym**
***Gender***	*0.2*	*Female*	*235*	*64*			
		*Male*	*322*	*68*			
***Marital status***	*0.046*	*Married*	*233*	*74*			
		*Others*	*334*	*58*			
**Race**	0.073	White Or Caucasian	401	95			
		Others	156	37			
**Religion**	0.85	Latter Day Saints	201	51			
		Others	356	81			
**Discharge disposition**	≤0.001	Home Or Self Care	348	77	2	1	1
		Others	209	55			
**Hospital services**	≤0.001	Cardiology	366	72	8	2	7
		Others	191	60			
**Insurance/finance class**	0.12	Medicare	347	73			
		Others	210	59			
**Prior 6-month ED frequency**	0.001	1	250	41	13	6	12
		Others	307	91			
**Age-at-Admission (AGE)**	≤0.001	≤ 83.5	475	129	3	8	5
		>83.5	82	3			
**Length of Stay (LOS)**	≤0.001	≤ 5	330	68			
		>5	227	64			
**Home proximity**	≤0.001	≤ 44	407	86			
		>44	150	46			
***Mean household income***	≤0.001	*≤68958.5*	*364*	*86*			
		*>68958.5*	*193*	*46*			
**Prior 6-month mean ED LOS**	≤0.001	≤0.235	382	110	11	13	13
		>0.235	175	22			

Variables included as risk factors in the final model are indicated in non-italic font, and variables excluded from the model are indicated in italics. The dataset used is the 2008–2012 derivation dataset. The count under Yes indicates the frequency of the variable value when readmissions occurred, and the coun under No indicates the frequency of the variable value when readmissions did not occur. G/W, Info and Sym are the relative ranks of the most significant variables (relative weight ≥ 0.001) using the GainRatioAttributeEval/Wrapper, InfoGainAtttributeEval, and SymmetricalUncertAttrbuteEval ranking strategies, respectively. G/W combines two strategies, because the relative ranks were equivalent using both approaches.

**Table 5:** Final status of numeric explanatory variables- laboratory variables

	**P-values**	**Mean**	**SD**	**G/W**	**Inf**	**Sym**
**Red cell distribution width (11.7-26.7)**	<0.0001	15.86	2.26	1	9	4
***Glucose, Serum or Plasma***	*<0.0001*	*111*	*43.55*			
***Urea Nitrogen, Serum or Plasma***	*<0.0001*	*35.47*	*22.23*			
**Sodium, Serum or Plasma**	<0.0001	136.2	4.54			
**Creatinine, Serum or Plasma**	<0.0001	1.52	1.05			
**Potassium, Serum or Plasma**	<0.0001	4.13	0.52			
**Hemoglobin (6.3-18.8)**	<0.0001	11.73	2.42	5	3	2
**Mean Platelet Volume**	<0.0001	8.91	1.08			
**Platelet**	<0.0001	250	114.28			
**Mean Corpuscular Hemoglobin**	<0.0001	29.23	3			
**Mean Corpuscular Volume**	<0.0001	89.61	7.27			
**Red Blood Cell (1.97-7.04)**	<0.0001	4.04	0.85	5	7	8
**Hematocrit (19–59.9)**	<0.0001	36	7.28	6	4	3
***White Blood Cell Count***	*<0.0001*	*8.05*	*3.03*			
**Mean Corpuscular Hgb Concentration**	<0.0001	32.6	1.52			
**Granulocyte%**	*<0.0001*	*72.58*	*10.49*			
**Basophil% (0–2.4)**	<0.0001	0.44	0.31	4	6	7
**Eosinophil%**	<0.0001	2.38	2.4			
**Lymphocyte%**	<0.0001	18.11	9.01			
**Monocyte%**	<0.0001	6.5	2.14			
***Granulocyte #***	*<0.0001*	*6.3*	*3.32*			
**Basophil #**	<0.0001	0.02	0.04			
**Lymph #**	<0.0001	1.42	0.74			
**Monocyte #**	<0.0001	0.53	0.23			
**Eosinophil #**	<0.0001	0.18	0.19			
**Protein, Total, Serum or Plasma**	<0.0001	6.73	0.83			
**Bilirubin, Total**	<0.0001	1.09	2.19			
**Albumin, Serum or Plasma**	<0.0001	3.49	0.55			
***Alanine Aminotransferase***	*<0.0001*	*54.32*	*76.02*			
***Aspartate Aminotransferase***	*<0.0001*	*66.43*	*96.51*			
***Alkaline Phosphatase***	*<0.0001*	*118.1*	*66.85*			
***B-Type Natriuretic Peptide***	*<0.0001*	*1477*	*129.56*			
**CharlsonIndex frequency**	<0.0001	4.66	2.64	9	11	9
***Excluded comorbidities***	*0.001*	*[0.2,4.8]*	*[0.45,2.45]*			
**Diseases of the musculoskeletal system and connective tissue**	<0.0001	0.35	0.69			
**Injury and poisoning**	<0.0001	0.35	0.66	12	10	11
**Last Reading Systolic Blood Pressure (LSBP)**	<0.0001	110.8	21.08			
**Last Reading Heart Rate (LHR)**	<0.0001	80.16	15.78			
**First Reading Weight KG (FW)**	<0.0001	91.36	31.92			
**Last Reading Weight KG (LW)**	<0.0001	88.87	46.21			
**Prior charlsonIndex frequency**	<0.0001	3.34	8.06	10	12	10

Variables included as risk factors in the final model are indicated in non-italic font, and variables excluded from the model are indicated in italics. The dataset used is the 2008–2012 derivation dataset. G/W, Info and Sym are the relative ranks of the most significant variables (relative weight ≥ 0.001) using the GainRatioAttributeEval/Wrapper, InfoGainAtttributeEval, and SymmetricalUncertAttrbuteEval ranking strategies, respectively. G/W combines two strategies, because the relative ranks were equivalent using both approaches. All ranked variables are listed with their ranges.

As a comparison, we used the LACE index approach to develop predictive models for readmissions [57] with our three complete derivation datasets, i.e. 2003–2012, 2008–2012, and 2009–2012. On our validation dataset, these three LACE index predictive models resulted in c-statistics of 63.0%, 65.1%, and 64.3% respectively. Moreover, when we utilized various other classifiers reported in the literature for readmission [12,21,32,33] to develop predictive models using our datasets, we acquired worse performance than our proposed voting classifier, with the c-statistics ranging from 58.3% to 68.1% on our validation dataset.

### Risk factor analysis step

Tables 4 and 5 show the results from the above statistical and ranking strategies to identify significant risk factors among the 67 potential explanatory variables analyzed using the complete 2008–2012 derivation dataset. Among the 25 explanatory variables not selected in step one (indicated in italics in the tables), none were identified as risk factors using the algorithm specified earlier (Algorithm 1- Overview of Approach subsection). Among the 42 explanatory variables selected in step one (indicated in normal font in the tables), none were removed as risk factors using this algorithm.

#### Weak risk factors

Of the 42 risk factors, race, religion, and insurance/finance class variables had weak statistical significance but were included in the step one selection process, and the classifier performed better when they were included as features. Therefore, these three variables were identified as weak risk factors.

#### Regular and strong risk factors

The remaining 39 variables were statistically significant predictive variables and were included in the step one selection process. Among them, 13 were ranked as the most important classifier variables by the four ranking strategies, where GainRatioAttributeEval and Wrapper rankers acquired equal variable ranks. These strongest identified risk factors were discharge disposition, discretized age, and anemia-related factors (RCDW, HGB, RBC, HCT, and BASO%). The other strong risk factors were hospital service, CharlsonIndexF, injury and poisoning (CCS diagnosis category 16), and the prior 6-month variables of CharlsonIndexF, FreqED and EDLOS.

#### Details for risk factors of special interest

Here, details are provided for the following risk factors: weak risk factors, strong non-discretized risk factors, and discretized risk factors.

Regarding the weak risk factors, the highest readmission rates were 39.9%, 33.3%, and 29.4% for the Asian race, the Protestant religion, and commercial insurance respectively.

Regarding strong non-discretized risk factors, discharge disposition was ranked first by two rankers and second by two rankers, indicating it was likely the most important risk factor in this model. Discharge to a rehabilitation facility was associated with the highest readmission rate (61.1%). Five laboratory variables were identified as strong risk factors: RCDW, HGB, RBC, HCT, and BASO%. These risk factors were frequently identified as one of the top five risk factors. Most of the readmitted patients had values within the ranges of 12.53-20.87, 7.19-14.28, 2.92-5.19, 21.77-43.92, and 0–1.21 respectively, and the associated highest readmission rates (20.4%-22.3%) were associated with laboratory values in the range of 14.2-15.87, 8.96-9.85, 3.58-3.90, 27.31-30.08, and 0–0.14 respectively.

**Figure 5:**
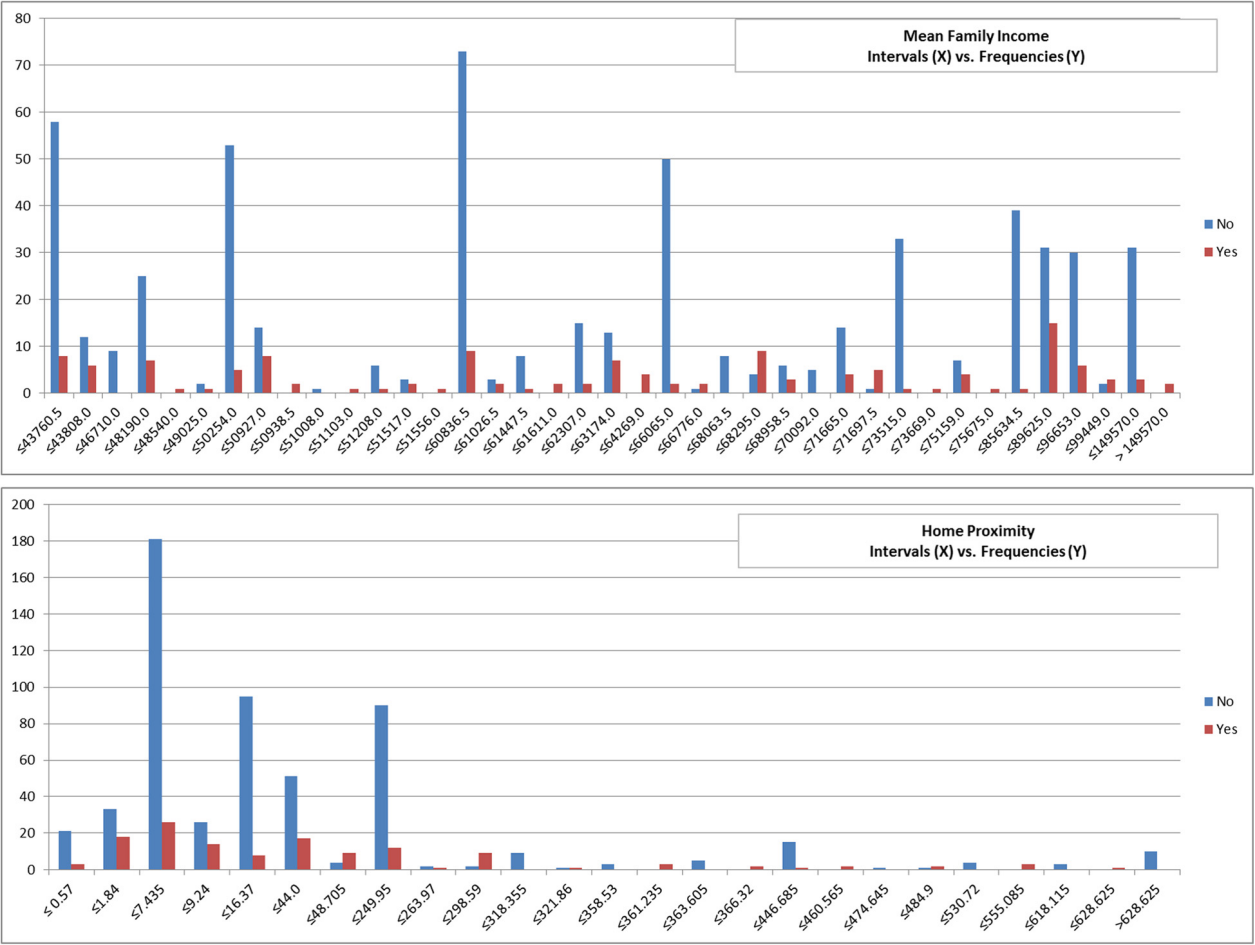
**Readmissions stratified according to discretized mean family income and proximity.** The X axis stands for the intervals of the variable and the Y axis stands for the related frequencies.

**Figure 6:**
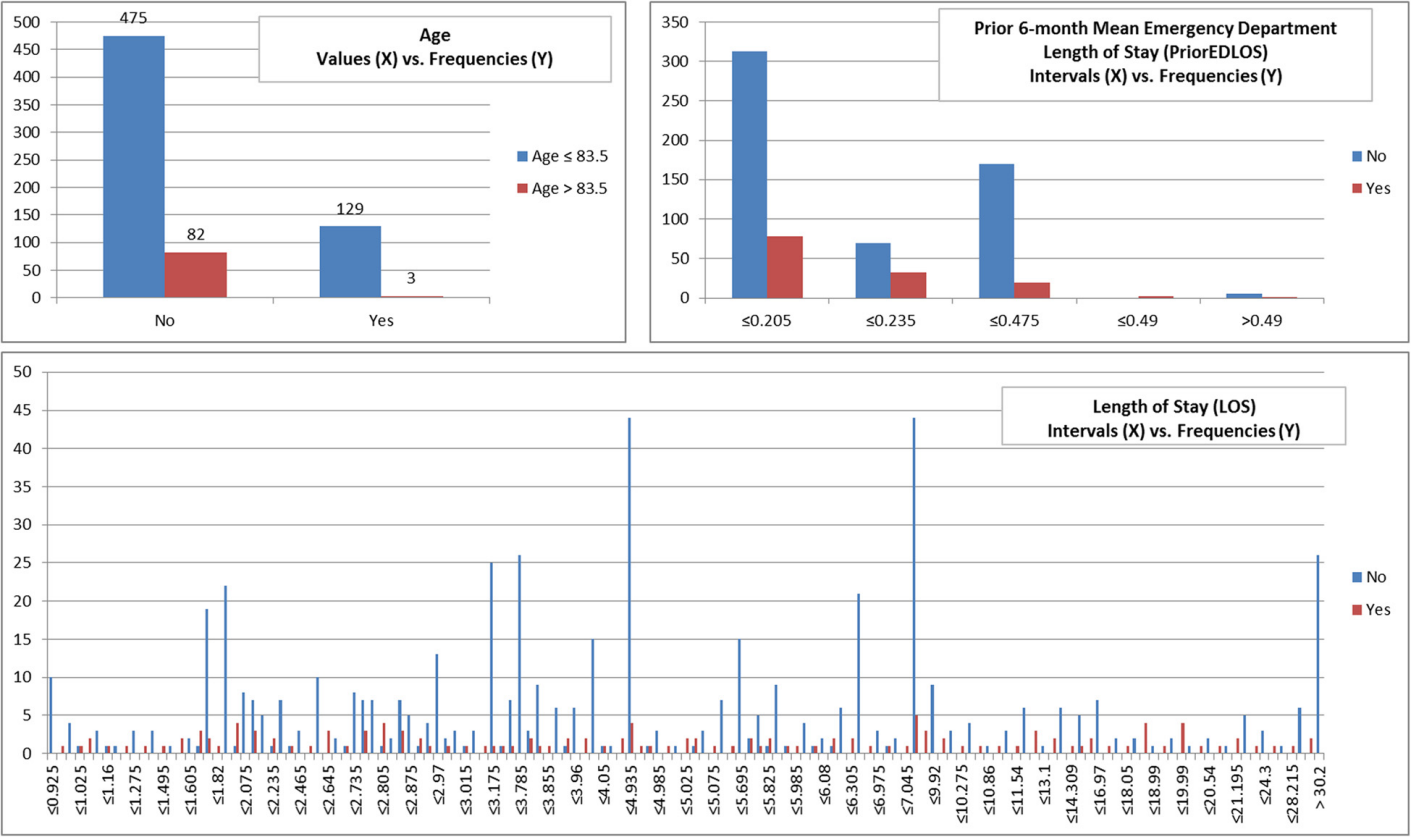
**Readmissions stratified according to discretized age, length of stay (LOS) and prior 6-month mean emergency department length of stay (PriorEDLOS).** The X axis stands for the values/intervals of the variable and the Y axis stands for the related frequencies.

The other strong risk factors, which ranked frequently from 8 to 13, are summarized as follows. For hospital service, the highest readmission rate (32.7%) was for patients cared for by cardiothoracic surgery. With regard to prior ED utilization, the highest readmission rate (42.9%) was for patients who were seen at the ED at least 6 times in the prior 6 months. The highest readmission rates (range 20.2%-21.8%) for CharlsonIndexF, CCS category 16 (injury and poisoning), and prior 6-month CharlsonIndexF variables were associated with frequencies of 2–5, 0–2, and 0–5 respectively. Among patients with an ICD-9 diagnosis in the injury and poisoning CCS category, the most common reason for that categorization was a diagnosis of hypoxemia (present in 23.4% of patients with the CCS category).Figures 5 and 6 show the frequencies of each discretized risk factor, with intervals determined according to the CACC-D discretization algorithm. With respect to age, the highest readmission rate (22.5%) occurred among patients aged 68–75. Most of the readmissions were associated with a LOS of between 10 to 30 days, where the readmission rate was 35.8%. With regard to home proximity, the highest readmission rate (23.5%) was for patients residing > 44 miles away from the hospital. With regard to PriorEDLOS, the highest readmission rate (22.4%) was for patients whose ED stays over the past 6 months averaged ≤ 0.235 days per stay. Among these discretized risk factors, age and PriorEDLOS were strong risk factors, which were often ranked third and eleventh respectively.

### Evaluation of potential for overfitting

Given the relatively small sample size and the large number of features evaluated, we evaluated for the potential for overfitting by testing the model on a separate, unseen validation dataset separate from the derivation and primary validation datasets. This additional validation dataset consisted of 130 CHF admissions from July to October 2013, 19 of which resulted in a readmission (14.5% readmission rate), and 45.73% of which contained complete data. The performance characteristics of the best model remained strong when evaluated against this additional validation dataset, with an AUC of 79.0%, accuracy of 85.4%, sensitivity of 55.3%, specificity of 88.2%, PPV of 44%, and NPV of 90.4%. Moreover, the LACE index predictive model [57] achieved an AUC of 60% on this dataset, and the approaches used by [12,21,32,33] resulted in an AUC of 53.1%-62.2%. This strong performance of the predictive model on a separate validation dataset indicates that overfitting was not a problem.

Of note, as with any predictive model [10], the performance of the model described in this manuscript is dependent on the underlying relationship between the explanatory variables and the outcome of interest remaining stable over time. Given the constantly evolving nature of patient care practices, this and other clinical predictive models need to be re-evaluated and re-tooled over time to ensure their continued relevance and predictive ability.

## Discussion

### Summary of findings

In this study, a three-step approach to predictive analytics was proposed and piloted on an operational clinical dataset to develop predictive models for CHF readmission. This approach adds to the healthcare literature by acknowledging the changing nature of operational data over time and systematically evaluating various temporal datasets. Moreover, many of the component techniques included in our proposed approach, such as voting classifiers, discretization, wrapper subset feature selection, and various ranking strategies, have not been applied to readmission predictive analytics in the past. Furthermore, to our knowledge, our proposed three-step approach to optimizing predictive analytics has never been applied to date in the healthcare domain. The resulting predictive model had a c-statistic of 86.8%.

Our findings are generally consistent with the prior literature in this field, with many of the same risk factors identified for CHF readmission, such as age, LOS, HGB, and HCT [33]. At the same time, discharge disposition, which was the highest ranked risk factor in our model, has not typically been identified as a top risk factor previously.

### Strengths and limitations of approach

An important strength is that the approach is generic and can be generalized to other problem spaces. As a second strength, the model had strong performance, with an 86.8% c-statistic, despite the unseen validation dataset containing all records, including 52.7% of records with missing data. Third, as discussed in the Future Directions section below, the model is designed for operational use, utilizing data that are operationally available, including data that change over time and are frequently missing. Fourth, our approach to risk factor selection utilizes not only statistical methods but also classifier feature selection with ranking strategies. By using multiple independent methods for risk factor selection, our approach increases confidence in the absolute and relative importance of the risk factors that are identified as important by multiple methods. Finally, our approach is based on the use of open-source, readily available tooling and can be replicated by others at minimal cost.

With regard to limitations, the study was conducted in a single, relatively small academic health system. Consequently, the results of the study will need to be replicated at other institutions to verify external validity. Second, the approach has not yet been applied to other problem spaces. Therefore, additional studies utilizing this approach will be needed to evaluate its applicability to other domains.

### Implications

In this study, a systematic, three-step approach to predictive analytics has been applied in the domain of CHF readmissions and validated to produce high-performing predictive models. In reducing CHF readmissions specifically, and potentially readmissions in general, our approach could potentially support the development of targeted interventions for addressing this important cause of morbidity, mortality, and excess healthcare costs. The potential for the development of such interventions is discussed below under Future Directions. Moreover, the approach is generic in nature and could potentially be applied in many other areas of health care where predictive analytics could help improve the efficiency and effectiveness of patient care.

### Future directions

Based on the results of this study, we are exploring how the predictive model could be used prospectively in an operational clinical setting. Almost all data points used in the model development are available at the time of hospitalization. Moreover, the few data points that would not be available in the EHR system prior to discharge (final discharge disposition, length of stay, and discharge diagnoses) could either be manually entered or inferred. For example, the final discharge disposition could be inferred from the anticipated discharge disposition as documented by the case manager, length of stay could be inferred using the anticipated discharge date documented by the case manager, and problem list entries could be used as a surrogate for discharge diagnoses.

Using this data, the model can provide a visit-specific probability of readmission that could be used to drive discharge planning and follow-up. For example, patients identified as being at higher risk of readmission could be followed more closely following discharge, for example through daily check-ins by a care manager. The final predictive model is embodied in a Java package that could be readily embedded within an information system to calculate patient-specific readmission probabilities. This information could be made available to care givers through a separate, stand-alone system. Alternatively, this information could potentially be made available as a part of the EHR system using a system integration approach such as that proposed by Zhang *et al.*[58].

All aspects of the three-step model development process could be automated, so that the full development process is automatically repeated periodically. Moreover, to reduce the required computational time, the model re-development process could be configured to use only those methods identified as being most effective in earlier iterations of the full three-step process (e.g., for discretization and classification). We are currently in the process of implementing this automated approach.

Moving forward, we believe that the proposed approach to predictive analytics should be applied to other clinical problems and datasets. We are currently in the process of conducting such analyses, and preliminary indications look promising. We also are working on improving our approach, for example by developing systematic approaches for grouping similar values for categorical variables and investigating enhanced methods for imputing missing values.

## Conclusion

This paper proposed, implemented, and evaluated a systematic, three-step approach to predictive analytics for health care. The approach performs well when applied to the prediction of CHF readmissions and is designed to be generalizable to other problem domains. We anticipate that this approach will contribute to the further use of predictive analytics to improve health and health care.

## Competing interests

The authors have no competing interests to declare.

## Authors’ contributions

SEA led the design and conduct of the study, as well as the initial preparation of the manuscript. KK oversaw the design and conduct of the study, as well as the preparation of the manuscript. MZ prepared the analysis datasets, BEB provided clinical guidance on the selection of potential explanatory variables, and both MZ and BB contributed to the review and editing of the manuscript. All authors read and approved the final manuscript.

## Pre-publication history

The pre-publication history for this paper can be accessed here:

http://www.biomedcentral.com/1472-6947/14/41/prepub

## References

[B1] Readmissions Reduction Program http://www.cms.gov/Medicare/Medicare-Fee-for-Service-Payment/AcuteInpatientPPS/Readmissions-Reduction-Program.html

[B2] Jencks SF Williams MV Coleman EA Rehospitalizations among patients in the Medicare fee-for-service program N Engl J Med 2009 360 14 1418 1428 19339721 10.1056/NEJMsa0803563

[B3] Allaudeen N Schnipper JL Orav EJ Wachter RM Vidyarthi AR Inability of providers to predict unplanned readmissions J Gen Intern Med 2011 26 7 771 776 21399994 10.1007/s11606-011-1663-3 PMC3138589

[B4] Allaudeen N Vidyarthi A Maselli J Auerbach A Redefining readmission risk factors for general medicine patients J Hosp Med 2011 6 2 54 60 20945293 10.1002/jhm.805

[B5] Amalakuhan B Kiljanek L Parvathaneni A Hester M Cheriyath P Fischman D A prediction model for COPD readmissions: catching up, catching our breath, and improving a national problem 2012 Perspectives: Journal of Community Hospital Internal Medicine 2(1) 10.3402/jchimp.v2i1.9915 PMC3714087 23882354

[B6] Garcia-Perez L Linertova R Lorenzo-Riera A Vazquez-Diaz JR Duque-Gonzalez B Sarria-Santamera A Risk factors for hospital readmissions in elderly patients: a systematic review QJM 2011 104 8 639 651 21558329 10.1093/qjmed/hcr070

[B7] Halfon P Eggli Y van Melle G Chevalier J Wasserfallen JB Burnand B Measuring potentially avoidable hospital readmissions J Clin Epidemiol 2002 55 6 573 587 12063099 10.1016/s0895-4356(01)00521-2

[B8] Hasan O Meltzer DO Shaykevich SA Bell CM Kaboli PJ Auerbach AD Wetterneck TB Arora VM Zhang J Schnipper JL Hospital readmission in general medicine patients: a prediction model J Gen Intern Med 2010 25 3 211 219 20013068 10.1007/s11606-009-1196-1 PMC2839332

[B9] Howell S Coory M Martin J Duckett S Using routine inpatient data to identify patients at risk of hospital readmission BMC Health Serv Res 2009 9 96 96 19505342 10.1186/1472-6963-9-96 PMC2700797

[B10] Kansagara D Englander H Salanitro A Kagen D Theobald C Freeman M Kripalani S Risk prediction models for hospital readmission: a systematic review JAMA 2011 306 15 1688 1698 22009101 10.1001/jama.2011.1515 PMC3603349

[B11] Khawaja FJ Shah ND Lennon RJ Slusser JP Alkatib AA Rihal CS Gersh BJ Montori VM Holmes DR Bell MR Curtis JP Krumholz HM Ting HH Factors associated with 30-day readmission rates after percutaneous coronary intervention Arch Intern Med 2012 172 2 112 117 22123752 10.1001/archinternmed.2011.569 PMC3688066

[B12] Lee EW Selecting the best prediction model for readmission J Prev Med Public Health 2012 45 4 259 266 22880158 10.3961/jpmph.2012.45.4.259 PMC3412989

[B13] Lichtman JH Leifheit-Limson EC Jones SB Watanabe E Bernheim SM Phipps MS Bhat KR Savage SV Goldstein LB Predictors of hospital readmission after stroke: a systematic review Stroke 2010 41 11 2525 2533 20930150 10.1161/STROKEAHA.110.599159 PMC3021413

[B14] Silverstein MD Qin H Mercer SQ Fong J Haydar Z Risk factors for 30-day hospital readmission in patients ≥65 years of age Proc (Bayl Univ Med Cent) 2008 2008 363 372 18982076 10.1080/08998280.2008.11928429 PMC2566906

[B15] Van Walraven C Bennett C Jennings A Austin PC Forster AJ Proportion of hospital readmissions deemed avoidable: a systematic review CMAJ 2011 183 7 E391 E402 21444623 10.1503/cmaj.101860 PMC3080556

[B16] Walraven CV Wong J Forster A LACE+ index: extension of a validated index to predict early death or urgent readmission after hospital discharge using administrative data Open Med 2012 6 3 e80 e90 23696773 PMC3659212

[B17] Coleman EA Min SJ Chomiak A Kramer AM Posthospital care transitions: patterns, complications, and risk identification Health Serv Res 2004 39 5 1449 1465 15333117 10.1111/j.1475-6773.2004.00298.x PMC1361078

[B18] Choubey SK Deogun JS Raghavan VV Sever H A comparison of feature selection algorithms in the context of rough classifiers 1996 2 1122 1128

[B19] Lazar C Taminau J Meganck S Steenhoff D Coletta A Molter C de Schaetzen V Duque R Bersini H Nowe A A survey on filter techniques for feature selection in gene expression microarray analysis IEEE/ACM Trans Comput Biol Bioinform 2012 9 4 1106 1119 22350210 10.1109/TCBB.2012.33

[B20] Molina LC Belanche L Nebot A Feature selection algorithms: a survey and experimental evaluation ICDM '02 Proceedings of the 2002 IEEE International Conference on Data Mining 2002 USA: IEEE Computer Society 306 313

[B21] Agarwal J Predicting Risk of Re-hospitalization for Congestive Heart Failure Patients 2012 Masters of Science: University of Washington

[B22] Au AG McAlister FA Bakal JA Ezekowitz J Kaul P van Walraven C Predicting the risk of unplanned readmission or death within 30 days of discharge after a heart failure hospitalization Am Heart J 2012 164 3 365 372 22980303 10.1016/j.ahj.2012.06.010

[B23] Brand C Sundararajan V Jones C Hutchinson A Campbell D Readmission patterns in patients with chronic obstructive pulmonary disease, chronic heart failure and diabetes mellitus: an administrative dataset analysis Intern Med J 2005 35 5 296 299 15845113 10.1111/j.1445-5994.2005.00816.x

[B24] Coffey RM Misra A Barrett M Andrews RM Mutter R Moy E Congestive heart failure: who is likely to be readmitted? Med Care Res Rev 2012 69 5 602 616 22653415 10.1177/1077558712448467

[B25] Gronda E Mangiavacchi M Andreuzzi B Municino A Bologna A Schweiger C Barbieri P A population-based study on overt heart failure in Lombardy (survey of hospitalization in 1996 and 1997) Ital Heart J 2002 3 2 96 103 11926018

[B26] Hammill BG Curtis LH Fonarow GC Heidenreich PA Yancy CW Peterson ED Hernandez AF Incremental value of clinical data beyond claims data in predicting 30-day outcomes after heart failure hospitalization Circulation Cardiovascular quality and outcomes 2011 4 1 60 67 21139093 10.1161/CIRCOUTCOMES.110.954693

[B27] Harjai KJ Thompson HW Turgut T Shah M Simple clinical variables are markers of the propensity for readmission in patients hospitalized with heart failure Am J Cardiol 2001 87 2 234 237 A239 11152851 10.1016/s0002-9149(00)01328-x

[B28] Jiang W Alexander J Christopher E Kuchibhatla M Gaulden LH Cuffe MS Blazing MA Davenport C Califf RM Krishnan RR O'Connor CM Relationship of depression to increased risk of mortality and rehospitalization in patients with congestive heart failure Arch Intern Med 2001 161 15 1849 1856 11493126 10.1001/archinte.161.15.1849

[B29] Joynt KE Jha AK Who has higher readmission rates for heart failure, and why? Implications for efforts to improve care using financial incentives Circulation Cardiovascular quality and outcomes 2011 4 1 53 59 21156879 10.1161/CIRCOUTCOMES.110.950964 PMC3050549

[B30] Kossovsky MP Sarasin FP Perneger TV Chopard P Sigaud P Gaspoz J-M Unplanned readmissions of patients with congestive heart failure: do they reflect in-hospital quality of care or patient characteristics? Am J Med 2000 109 5 386 390 11020395 10.1016/s0002-9343(00)00489-7

[B31] Krumholz H Normand S-L Keenan P Lin Z Drye E Bhat K Wang Y Ross J Schuur J Stauffer B Bernheim S Epstein A Herrin J Federer J Mattera J Wang Y Mulvey G Schreiner G Hospital 30-day heart failure readmissionmeasure:methodology Centers for Medicare & Medicaid Services (CMS) 2008

[B32] Natale J Wang S Taylor J A Decision Tree Model for Predicting Heart Failure Patient Readmissions Proceedings of the 2013 Industrial and Systems Engineering Research Conference 2–13 2 13

[B33] Ross JS Mulvey GK Stauffer B Patlolla V Bernheim SM Keenan PS Krumholz HM Statistical models and patient predictors of readmission for heart failure: a systematic review Arch Intern Med 2008 168 13 1371 1386 18625917 10.1001/archinte.168.13.1371

[B34] Wong EL Cheung AW Leung MC Yam CH Chan FW Wong FY Yeoh EK Unplanned readmission rates, length of hospital stay, mortality, and medical costs of ten common medical conditions: a retrospective analysis of Hong Kong hospital data BMC Health Serv Res 2011 11 149 21679471 10.1186/1472-6963-11-149 PMC3146405

[B35] Zai AH Ronquillo JG Nieves R Chueh HC Kvedar JC Jethwani K Assessing hospital readmission risk factors in heart failure patients enrolled in a telemonitoring program International journal of telemedicine and applications 2013 2013 305819 23710170 10.1155/2013/305819 PMC3655587

[B36] Ibrahim JG Chu H Chen MH Missing data in clinical studies: issues and methods J Clin Oncol 2012 30 26 3297 3303 22649133 10.1200/JCO.2011.38.7589 PMC3948388

[B37] Little RJ D'Agostino R Cohen ML Dickersin K Emerson SS Farrar JT Frangakis C Hogan JW Molenberghs G Murphy SA Neaton JD Rotnitzky A Scharfstein D Shih WJ Siegel JP Stern H The prevention and treatment of missing data in clinical trials N Engl J Med 2012 367 14 1355 1360 23034025 10.1056/NEJMsr1203730 PMC3771340

[B38] Luengo J García S Herrera F On the choice of the best imputation methods for missing values considering three groups of classification methods Knowl Inform Syst 2011 32 1 77 108

[B39] Kittler J Hatef M Duin RPW Matas J On Combining Classifiers IEEE Trans Pattern Anal Mach Intell 1998 20 3 226 239

[B40] Torii M Hu Z Wu CH Liu H BioTagger-GM: a gene/protein name recognition system J Am Med Inform Assoc 2009 16 2 247 255 19074302 10.1197/jamia.M2844 PMC2649315

[B41] Wu Y Rosenbloom ST Denny JC Miller RA Mani S Guise DA Xu H Detecting Abbreviations in Discharge Summaries using Machine Learning Methods AMIA Annu Symp Proc: 2011; Chicago, IL 2011 PMC3243185 22195219

[B42] Lustgarten JL Gopalakrishnan V Grover H Visweswaran S Improving Classification Performance with Discretization on Biomedical Datasets AMIA 2008 Symposium Proceedings 2008 445 449 PMC2656082 18999186

[B43] Lustgarten JL Visweswaran S Gopalakrishnan V Cooper GF Application of an efficient Bayesian discretization method to biomedical data BMC Bioinformatics 2011 12 309 21798039 10.1186/1471-2105-12-309 PMC3162539

[B44] The U.S. Census Bureau http://www.census.gov/

[B45] Haversine formula http://en.wikipedia.org/wiki/Haversine_formula

[B46] Population Studies Center at the University of Michigan http://www.psc.isr.umich.edu/

[B47] Quan H Sundararajan V Halfon P Fong A Burnand B Luthi JC Saunders LD Beck CA Feasby TE Ghali WA Coding algorithms for defining comorbidities in ICD-9-CM and ICD-10 administrative data Medical care 2005 43 11 1130 1139 16224307 10.1097/01.mlr.0000182534.19832.83

[B48] Balas EA Austin SM Mitchell JA Ewigman BG Bopp KD Brown GD The clinical value of computerized information services. A review of 98 randomized clinical trials Arch Fam Med 1996 5 5 271 278 8620266 10.1001/archfami.5.5.271

[B49] Desai MM Stauffer BD Feringa HH Schreiner GC Statistical models and patient predictors of readmission for acute myocardial infarction: a systematic review Circulation Cardiovascular quality and outcomes 2009 2 5 500 507 20031883 10.1161/CIRCOUTCOMES.108.832949

[B50] Weka 3.6 http://www.cs.waikato.ac.nz/ml/weka/downloading.html

[B51] Garci¿a S Luengo J Saez JA Lopez V Herrera F A Survey of Discretization Techniques: Taxonomy and Empirical Analysis in Supervised Learning IEEE Transactions on Knowledge and Data Engineering 2012

[B52] Kurgan LA Cios KJ CAIM discretization algorithm IEEE Trans Knowl Data Eng 2004 16 2 145 153

[B53] Tsai C-J Lee C-I Yang W-P A discretization algorithm based on Class-Attribute Contingency Coefficient Inform Sci 2008 178 3 714 731

[B54] Keel Software http://sci2s.ugr.es/keel/algorithms.php#discretization

[B55] Cessie SL Houwelingen JCV Ridge estimators in logistic regression J Roy Stat Soc C Appl Stat 1992 41 191 201

[B56] Demiröz G Güvenir HA Classification by voting feature intervals Machine Learning: ECML 97 1997 1224 85 92

[B57] Van Walraven C Dhalla IA Bell C Etchells E Stiell IG Zarnke K Austin PC Forster AJ Derivation and validation of an index to predict early death or unplanned readmission after discharge from hospital to the community CMAJ 2010 182 6 551 557 20194559 10.1503/cmaj.091117 PMC2845681

[B58] Zhang M Velasco F Musser R Kawamoto K Enabling Cross-Platform Clinical Decision Support through Web-Based Decision Support in Commercial Electronic Health Record Systems: proposal and Evaluation of Initial Prototype Implementations AMIA 2013 PMC3900165 24551426

